# Proanthocyanidins attenuated liver damage and suppressed fibrosis in CCl4-treated rats

**DOI:** 10.1007/s11356-022-22051-7

**Published:** 2022-07-26

**Authors:** Maher A. Amer, Azza I. Othman, Mohamed A. EL-Missiry, Aya A. Farag, Maggie E. Amer

**Affiliations:** Zoology Department, Faculty of Science, Mansoura University, Mansoura, Egypt

**Keywords:** Liver fibrosis, Hepatic inflammation, Oxidative stress, Apoptosis DNA strand breaks, Glutathione

## Abstract

Liver damage and fibrosis are serious health problems without effective treatment. Proanthocyanidins (PAs) are flavonoids with several biological effects. We investigated the potential anti-fibrotic effect of proanthocyanidins on carbon tetrachloride (CCl_4_)-induced liver injury and fibrosis. Liver fibrosis was induced by oral administration of CCl_4_ three times a week for 5 and 9 weeks. PAs were daily administered in a dose of 500 mg/kg bw. Animals were divided into five groups: control groups, olive oil-treated group, Pas-treated group, CCl4-treated animals, and PAs + CCl4-treated rats. CCl4 and PAs were administered by gavage. Administration of CCl_4_ caused a significant elevation in alanine aminotransferase and aspartate aminotransferase activities, the concentration of alpha-2-macroglobulin, and bilirubin concentration. In addition, the protein and apolipoprotein contents were significantly decreased in the serum of CCl4-treated rats. These results were accompanied by histopathological alterations and increased inflammation, apoptosis, and DNA damage. Treatment with PAs caused remarkable regression of fibrosis and alpha-2-macroglobulin with improvement in histological characteristics of the liver after 5 and 9 weeks of intoxication. PAs could also maintain redox balance, evidenced by the prevention of lipid peroxidation and mitigation of the decrease in antioxidants. Treatment of intoxicated rats with PAs resulted in a significant decline in pro-inflammatory cytokines, including IL-6, IL-1β, and TNF-α in serum. This is associated with a remarkable decrease in apoptosis of hepatic cells shown by decreased levels of Bax, caspase-3, and -9, with increased Bcl-2. The protective effect of PAs was also evident by protecting DNA integrity in the intoxicated rats. PAs suppressed hepatic fibrosis, improved liver function and structure via modulating the interdependence between oxidative stress, inflammation, apoptosis, and DNA integrity in CCl_4_-treated rats.

## Introduction

Hepatic fibrosis is a serious consequence of liver toxicity and might develop into liver cirrhosis without proper intervention. Besides the high mortality rate worldwide, it imposes an economic burden on all societies. Despite the appearance of many reports describing the etiology of hepatic fibrosis (Shan et al. 2019), the effective remedies to inhibit its progression require further research (Friedman and Pinzani 2022). Searching for a natural preventive agent to interfere with fibrogenesis is vital to avoid side effects and drug-drug interactions. Therefore, natural flavonoids from plant sources are highly appreciated approaches to treat liver damage and fibrosis (Latief and Ahmad 2017; Pan et al. 2020).

The cellular and molecular mechanisms described to explain fibrosis and its inhibition include oxidative stress, inflammation, and apoptosis of hepatic cells (Kisseleva and Brenner 2021). Mitochondrial functional activities are associated with controlling apoptosis, which were decreased in experimental liver fibrosis in mice (Sun et al. 2018). Persistent inflammation in the liver is the major factor in fibrosis initiation and progression and is associated with the activation of macrophages in many liver diseases (Calvente et al. 2019). Oxidative stress and inflammation are associated events and are pathogenic processes in liver diseases under severe etiology (Reyes-Gordillo et al. 2017).

A large number and variety of plant-derived products have remarkable preventive effects on liver fibrosis. This includes alkaloids, terpenoids, glycosides, and coumarins (Ma et al. 2020). Various plant-derived flavonoids from cranberry showed pronounced inhibitory effects on the development of liver fibrosis, including flavonoids, flavonol glycosides, anthocyanins, and PAs (Shi et al. 2021). PAs are oligomers or polymers of monomeric flavan-3-ols produced naturally by flavonoid synthesis (Rauf et al. 2019) and synthetically synthesized (Alejo-Armijo et al. 2020). The principal components of PAs include catechin and epicatechin (Dong 2015). Fruits (including berries), nuts, and spices are rich sources of PAs (Krenn et al. 2007; Alejo-Armijo et al. 2020). Pas have remarkable biological effects such as antioxidant (Lai et al. 2018), immunomodulatory (Rauf et al. 2019), and anti-apoptotic (Yin et al. 2017) in several conditions. In addition, PAs play a key role in modulating signaling transduction pathways involved in oxidative stress to perform protective functions (Yang et al. 2018; Gonzalez-Quilen et al. 2020). The biomedical properties of PAs also include antimicrobial, cardiovascular protection, neuroprotection, metabolism-regulation (Zeng et al. 2020), and maintaining the intestinal barrier function with modulation of intestinal microbiota (Gonzalez-Quilen et al. 2020). Toxicological studies in vitro and in vivo showed a high safety profile with no observable toxicity to humans (Yang et al. 2018; Zeng et al. 2020).

Until the current study, there are no reports on the effects of PAs on chronic liver injuries and fibrosis, including the underlying pathways of action. Therefore, the bioactivities of PAs provoked the interest. However, the downregulating effect of PAs on ovarian (Zhou et al. 2021) and heart (Rathinavel et al. 2018) fibrosis was recently reported. Moreover, procyanidin B2, a dimer type of proanthocynanidins, suppressed the activation of hepatic stellate cells and angiogenesis during liver fibrosis (Feng et al. 2019) and declined the inflammatory response and apoptosis against carbon tetrachloride (CCl_4_)-induced acute liver injury in mice (Yang et al. 2015).

In the current study, the protective effect of PAs against liver damage and development of fibrosis was studied in relation to the interdependence of oxidative stress, inflammation, apoptosis, and DNA integrity.

## Materials and methods

### Experimental chemicals

Carbon tetrachloride (CCl_4_) was obtained from BDH Laboratory supplies Poole, BH15 1TD England. PAs were provided by Sigma-Aldrich (St. Louis, MO, USA). Olive oil was obtained from a local source.

### Experimental animals

The Egyptian Vaccine Company (VACSERA, Giza, Egypt), supplied adult male Wistar rats (100–120 g). After a week of acclimatization, animals were housed in stainless steel cages under normal laboratory conditions with a 12 h light/dark cycle, 22 ± 2 °C, and 45–50% humidity. The experimental protocol to treat rats was performed according to the guidelines and approved by the Institutional Animal Ethics Committee (IAEC) of Mansoura University (DZ19005).

### Induction of chronic liver fibrosis and animal treatment

Liver fibrosis was induced in rats by oral administration of CCl_4_ dissolved in olive oil (V/V) at a dose of 2 mL/kg body weight orally (Liu et al. 2018) three times a week for 5 and 9 weeks. PAs was administered by gavage at 500 mg/kg body weight dose (Ding et al. 2013). Control groups were treated with olive oil (2 mL/kg body weight). No mortality was reported in animals because of CCl_4_ treatment.

### Experimental design

Animals were divided into five groups of 12 rats each, as follows.

1. Control groups (Cont): rats did not receive treatment.
2. Olive oil-treated groups (O oil): rats were orally administered olive oil alone (2 mL/kg bw) each day for 5 and 9 weeks.
3. PA groups (PAs): rats were orally administered (PAs) a 500 mg/kg bw dose daily for 5 and 9 weeks.
4. Carbon tetrachloride-treated groups (CCl_4_): rats were orally administered CCl_4_ dissolved in olive oil (1:1) each other day for 5 and 9 weeks.
5. PAs + CCl_4_-treated group (PAs + CCl_4_): rats were orally administered (PAs) (500 mg/kg bw) daily for 1-week prior CCl_4_ treatment, then PAs and CCl_4_ were administered concurrently every other day at the same doses administered to the third and fourth groups for 5 and 9 weeks.

### Sample collection

After 5 and 9 weeks of CCl_4_ treatment, rats were fasted overnight and sacrificed 24 h after the last treatment. Blood samples were collected in clean centrifuge tubes, then left to clot, then centrifuged at 1500 rpm for 15 min at 4 °C to obtain serum. The sera were frozen at − 20 °C for the biochemical analysis. Rat liver was removed, and part of the liver was weighed and homogenized in ice-cold 0.01 M Tris–HCl buffer (pH 7.4) using a Potter–Elvehjem glass-Teflon homogenizer. The homogenates were centrifuged at 1500 rpm, and the supernatant was kept at -20 °C till biochemical assays. For histopathological studies, other parts of liver tissue were stored in neutral formalin (10%).

### Biochemical analysis

Alanine aminotransferase (ALT) and aspartate aminotransferase (AST) activities, total bilirubin, and total protein contents were estimated colorimetry in serum using kits purchased from (Biodiagnostic Co., Dokki, Giza, Egypt), according to the kit’s instructions using a UV/Vis spectrophotometer (Model OPTIZEN POP, Mecasys Co., Ltd., Daejeon, Korea) to measure the absorbance of the solution at 505 nm (490–520 nm) for ALT and AST, 535 nm (530–540 nm) for total bilirubin and 550 nm (520–570 nm) for total protein.

Alpha-2-macroglobulin (A2M) and apolipoprotein (APOA1), using ELISA kits purchased from Abnova (Taipei, Taiwan) (Catalog No. KA1931) and CUSABIO (Baltimore, Maryland, USA) (Catalog No. BCSB–E08105r), respectively.

Malondialdehyde (MDA), glutathione peroxidase (GPx), glutathione reductase (GR), and reduced glutathione (GSH) were estimated colorimetry in the liver homogenate, following the instructions of the kits purchased from (Biodiagnostic Co., Dokki, Giza, Egypt) using a UV/Vis spectrophotometer (Model OPTIZEN POP, Mecasys Co., Ltd., Daejeon, Korea) measuring the absorbance of the solution at (534 nm) for MDA, (340 nm) for GPx, (340 nm) for GR, and (405 nm) for GSH.

In addition, interleukin 1 beta (IL-1β), interleukin 6 (IL-6), and tumor necrosis factor-alpha (TNF-a) levels in tissue were assessed using ELISA kits which were obtained from MyBioSource (San Diego, California, USA) (Catalog No. MBS825017), (Catalog No. MBS012805), and Rat TNF Alpha PicoKine™ ELISA Kit (Catalog No. MBS175904).

The levels of Bax and Bcl-2 in the liver were determined using rat ELISA kits provided by MyBioSource (San Diego, California, USA) (Catalog No MBS935667-48 T), (Catalog No MBS704498), respectively. The levels of caspase-3 and -9 in the liver were determined using rat ELISA kits provided by CUSABIO (Baltimore, Maryland, USA) (Catalog No. CSB-E08857r) and MyBioSource (San Diego, California, USA) (Catalog No. MBS765858), respectively.

### Single-cell gel electrophoresis (Comet assay)

DNA strand damage in the hepatocytes was estimated using the alkaline single-cell gel electrophoresis assay described previously (Singh 2000). Comet’s tail length was measured from the middle of the nucleus to the end of the tail. Using CASP software, tail length, tail moment, and percentage DNA in a tail were calculated (Sadeeshkumar et al. 2019).

### Histopathological examination

Portions of liver tissue were immediately fixed in 10% neutral formalin solution, dehydrated, cleared in xylene, embedded in paraffin wax, then sectioned at 5 µm, and stained with hematoxylin and eosin. The stained sections were examined and photographed under a light microscope (Leica microscope, Germany) to detect histopathological changes (Drury et al. 1967).

### Measurements of fibrosis in the liver

Paraffin-embedded liver tissue Sects. (5 to 7 mm) were stained with Masson’s trichrome to measure hepatic fibrosis. The sections were examined under a light microscope (Leica microscope, Germany) and photographed (DFC Leica camera). The blue stain reflects the extent of hepatic fibrosis and is employed to identify collagen fibers (Sheehan and Hrapchak 1980). Image analysis methods were performed using ImageJ software, version 1.48d (developed by Wayne Rasband, National Institutes of Health, Bethesda, Maryland, USA) (Rasband 1997–2014) and Java (64-bit) engine.

### Statistical analysis

Results are expressed as the means ± standard error of the mean (SEM) (*n* = 6). The statistical comparisons were performed using one-way ANOVA, followed by Tukey’s post hoc test. A significant difference was considered when the *P*-value was < 0.05. All statistical analyses were made using Graph Pad Prism 8.0 (Graph Pad Software Inc., San Diego, CA, USA).

## Results

### *PAs improved hepatic function parameters in CCL*_*4*_*-treated rats*

**Fig. 1:**
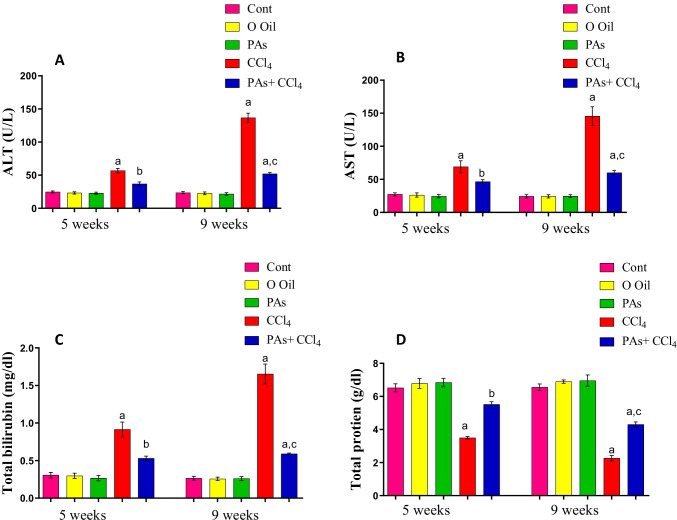
Effect of PAs and CCl_4_ on the activities of (A) alanine aminotransferase (ALT), (B) aspartate aminotransferase (AST) activities, and the levels of (C) total bilirubin and (D) total protein in serum of control and experimental rats. Data are expressed as mean ± SEM (*n* = 6 for each group). a, b, c Significant change at *P* < 0.05. a: Significance as compared with control, b: significance as compared with CCl_4_ group in 5 weeks, c: significance as compared with CCl_4_ group in 9 weeks. Cont, control; O oil, olive oil; PAs, PAs; CCl_4_, carbon tetrachloride

The induction of liver fibrosis by CCl_4_ caused a significant (*P* < 0.05) increase in the serum levels of liver function enzymes, including ALT and AST, as well as the total bilirubin contents compared to the control group, while total protein was significantly (*P* < 0.05) decreased in serum after 5 and 9 weeks of intoxication compared to the control group. The severity of changes in these clinical biomarkers was increased with time following CCl_4_ treatment. Administration of PAs into CCl_4_-treated rats caused a significant (*P* < 0.05) improvement in the levels of these biomarkers in serum compared with the CCL_4_-treated rats. Treatment with PAs or olive oil only displayed insignificant changes in these biomarkers in control rats after 5 and 9 weeks of treatment (Fig. 1).

### PAs improved A2M and APOA1 levels in the serum of fibrosis rat model

**Fig. 2:**
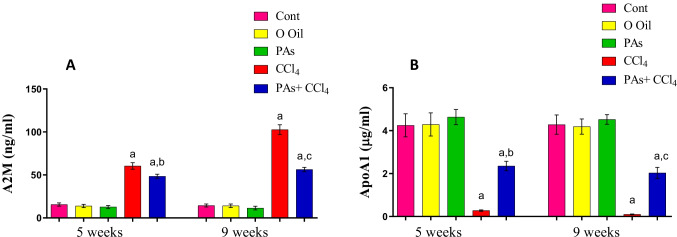
Effect of PAs and CCl_4_ on (A) alpha-2-macroglobulin (A2M) and (B) apolipoprotein (APOA1) in serum of the studied groups. Data are expressed as mean ± SEM (*n* = 6 for each group). a, b, c Significant change at *P* < 0.05. a: Significance as compared with control, b: significance as compared with CCl_4_ group in 5 weeks, c: significance as compared with CCl_4_ group in 9 weeks. Cont, control; O oil, olive oil; PAs, Pas; CCl_4_, carbon tetrachloride

The induction of liver fibrosis by CCl_4_ led to a significant (*P* < 0.05) increase in the serum levels of alpha-2-macroglobulin and a significant (*P* < 0.05) decrease in APOA1 in serum after 5 and 9 weeks of CCl_4_ treatment compared to the control group. In addition, administration of PAs in rats with CCl_4_-induced liver fibrosis resulted in a significant (*P* < 0.05) improvement in the levels of these biomarkers in serum compared with the CCL_4_-treated rats. However, oral administration with PAs or olive oil only showed insignificant changes in these biomarkers in control rats after 5 and 9 weeks of treatment (Fig. 2).

### PAs protected hepatic histological structures of the fibrosis rat model

**Fig. 3:**
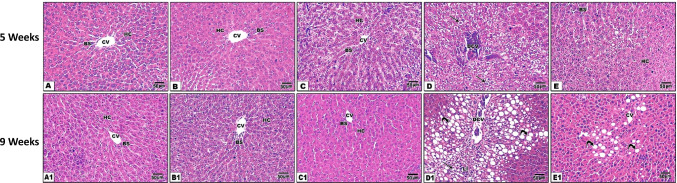
Photomicrographs of hematoxylin and eosin-stained hepatic tissue sections from all groups after 5 (A–E) and 9 (A1–E1) weeks of treatment. (A) Control, (B) olive oil, (C) PAs, (D) CCl_4_, (E) PAS + CCl_4_. (A1) Control, (B1) olive oil, (C1) PAs, (D1) CCl_4_, (E1) PAS + CCl_4_. Hepatocytes (HC), central vein (CV), blood sinusoid (BS), degenerated central vein (DCV), ballooning degeneration (arrow), leucocytic infiltration (LI), and steatosis (curved arrow). Sections (A–C) show normal hepatocytes arranged in cords around the CV, while (D) shows congestion of the CV, centrilobular hepatic degeneration (microvesicular fatty changes), and single-cell necrosis after 5 weeks of CCl_4_ treatment. (E) Section from the liver of PAs + CCl_4_ revealing a marked protection of hepatic histological structure evidenced by the decrease of hepatic fatty changes compared with (D). After 9 weeks of treatment with CCl_4_ (D1), severe histopathological changes were observed compared with (D) shown by a marked degree of advanced centrilobular hepatic fatty changes and increased fibroblastic cell proliferation. The protected groups (E and E1) showed a marked decrease in hepatic fatty changes and perivascular fibrosis compared with intoxicated rats (D–D1). H&E, × 200

Histopathological examination of liver tissues from control, olive oil, and PA groups revealed normal hepatic architecture with normal hepatocyte arrangement around the central vein. Liver section from CCl_4_-treated rats showed congestion and dilation of the central vein, ballooning degeneration, and steatosis. Treatment with PAs to CCl_4_-intoxicated rats for 5 weeks showed a marked amelioration of hepatic architecture and decreased hepatic steatosis (Fig. 3). After 9 weeks of CCl_4_ treatment, the liver section illustrated a marked degree of advanced periportal hepatic steatosis with increased fibroblastic cell proliferation and widened central vein, hemorrhage, and ballooning degeneration. Treatment with PAs showed marked amelioration of hepatic tissue accompanied by decreased steatosis and improving hepatic organization (Fig. 3).

**Fig. 4:**
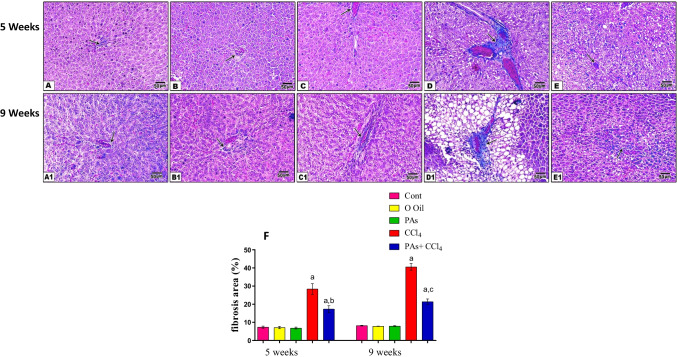
Masson trichrome-stained hepatic sections of different experimental groups (5 weeks, A–E) and (9 weeks (A1–E1) respectively. (A) Control, (B) olive oil, (C) PAs, (D) CCl_4_, (E) PAS + CCl_4_. (A1) Control, (B1) olive oil, (C1) PAs, (D1) CCl_4_, (E1) PAS + CCl_4_. The CCl_4_-treated animal (D and D1) shows increased fibrous connective tissue (arrow) between hepatocytes. Pretreatment with PAs (E) protected against the progression of hepatic fibrosis (arrow). (E) Quantificatfion of the fibrotic area at 5 and 9 weeks of treatment. Data are expressed as mean ± SEM (*n* = 6 for each group). a, b, c Significant change at *P* < 0.05. a: Significance as compared with control, b: significance as compared with CCl_4_ group in 5 weeks, c: significance as compared with CCl_4_ group in 9 weeks

Masson trichrome stain of liver sections (Fig. 4) shows a significant fibrotic area increase after 5 and 9 weeks of CCl_4_-treated rats. While, treatment of rats with PAs of CCl_4_ for 5 and 9 weeks displayed a significant (*P* < 0.05) lower fibrotic area than the CCl_4_-treated rats. The oral administration of PAs or olive oil for 5 and 9 weeks displayed an insignificant effect on the liver tissue fibrosis formation compared with the control group.

### PAs mitigated oxidative stress biomarkers in the liver of fibrosis rat model

**Fig. 5:**
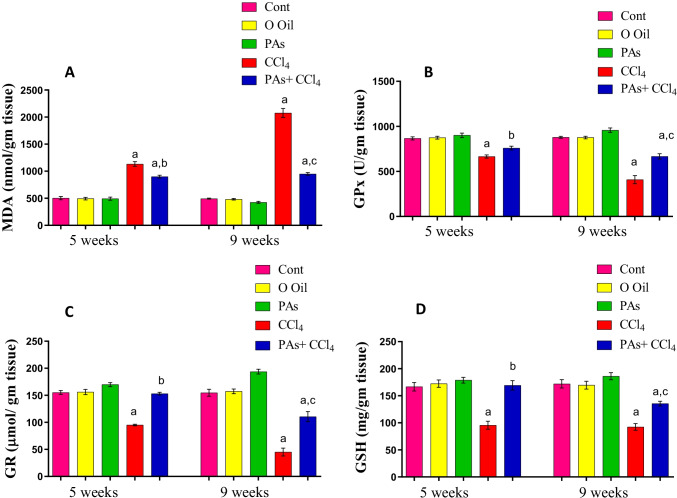
Effect of PAs and CCl4 on oxidative stress marker and antioxidants in control and experimental groups. (A) Malondialdehyde (MDA), (B) glutathione peroxidase (GPx), (C) glutathione reductase (GR), and (D) reduced glutathione (GSH) content in the studied groups. These parameters are expressed as the unit per gram wet tissue. Data are expressed as mean ± SEM (*n* = 6 for each group). a, b, c Significant change at *P* < 0.05. a: Significance as compared with control, b: significance as compared with CCl_4_ group in 5 weeks, c: significance as compared with CCl_4_ group in 9 weeks. Cont, control; O oil, olive oil; proanthocyanidins, PAs; CCl_4_, carbon tetrachloride

The induction of hepatic fibrosis by CCl_4_ showed a significant (*P* < 0.05) increase in hepatic MDA level as the end product of lipid peroxidation product in the liver after 5 and 9 weeks of CCl_4_ treatment compared to the control group. In contrast, concurrent administration of PAs in rats treated with CCl_4_ resulted in a significant (*P* < 0.05) reduction in hepatic MDA levels in the liver compared with the CCL_4_-treated rats. Treatment with PAs or olive oil alone did not affect MDA in livers in control rats (Fig. 5A).

### PAs improved antioxidants levels in the liver of the fibrosis rat model

Assessment of antioxidants liver rats treated with CCl_4_ showed a significant (*P* < 0.05) decrease in GSH content and GPx, and GR activities after 5 and 9 weeks of intoxication compared with the control rats. In addition, the loss of these antioxidants was significant (*P* < 0.05) with time laps following CCl_4_ treatment. However, oral administration of PAs into CCl_4_-induced liver fibrosis caused a significant (*P* < 0.05) improvement in the levels of these antioxidants in the liver compared with the CCL_4_-treated rats. No alternations were observed in hepatic GSH content and the activity of GPx and GR in rats that received PAs or olive oil alone compared with control rats (Fig. 5B–D).

### PAs improved pro-inflammatory cytokines in the liver of the fibrosis rat model

**Fig. 6:**
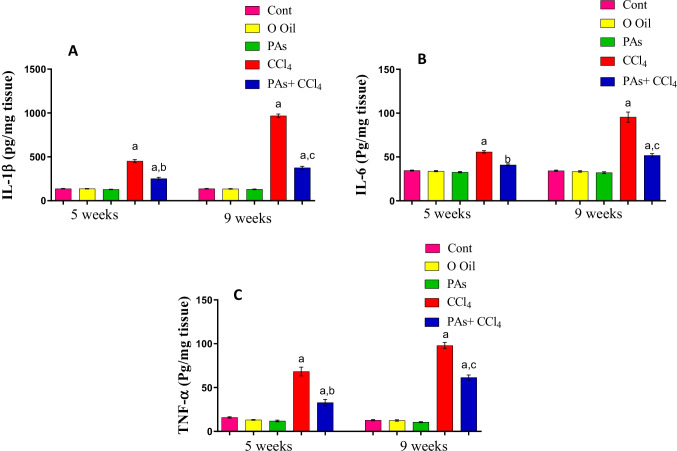
Effect of PAs and CCl_4_ on l pro-inflammatory cytokines. (A) Interleukin 1 beta (IL-1β), (B) interleukin 6 (IL-6), and (C) tumor necrosis factor-alpha (TNF-α) levels in the studied groups. These parameters are expressed as the unit per gram wet tissue. Data are expressed as mean ± SEM (*n* = 6 for each group). a, b, c Significant change at *P* < 0.05. a: Significance as compared with control, b: significance as compared with CCl_4_ group in 5 weeks, c: significance as compared with CCl_4_ group in 9 weeks. Cont, control; O oil, olive oil; proanthocyanidins, PAs; CCl_4_, carbon tetrachloride

The levels of pro-inflammatory cytokines (IL-1β, IL-6, and TNF-α) showed a significant (*P* < 0.05) increase after 5 and 9 weeks of hepatic fibrosis induction in rats compared with normal control rats. When PAs were administered to CCl_4_-intoxicated rats, the levels of these pro-inflammatory cytokines were significantly (*P* < 0.05) reduced compared with the CCL_4_-treated rats. The treatment with PAs or olive oil alone showed an insignificant difference in IL-1β, IL-6, or TNF-α level compared with the control rats (Fig. 6A–C).

### PAs modulated apoptotic regulating proteins in the liver of the fibrosis rat model

**Fig. 7:**
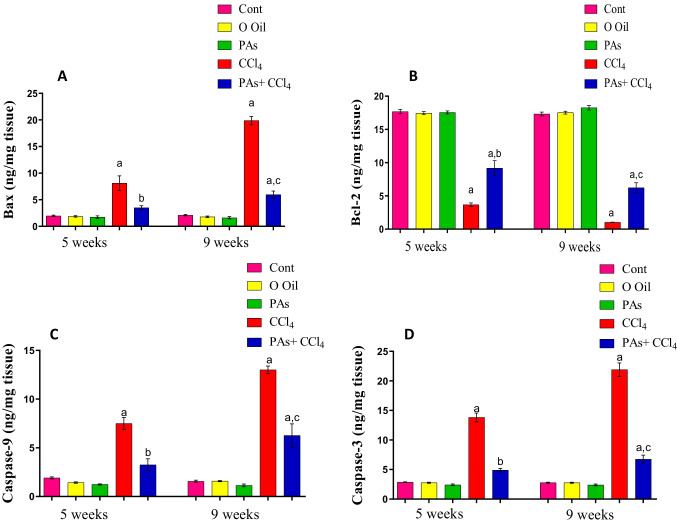
Effect of PAs and CCl4 on (A) pro-apoptotic proteins Bax and (B) anti-apoptotic protein Bcl-2, (C) caspase-9, and (D) caspase-3 levels in the liver of control and experimental groups. These parameters are expressed as the unit per gram wet tissue. Data are expressed as mean ± SEM (*n* = 6 for each group). a, b, c Significant change at *P* < 0.05. a: Significance as compared with control, b: significance as compared with CCl_4_ group in 5 weeks, c: significance as compared with CCl_4_ group in 9 weeks. Cont, control; O oil, olive oil; proanthocyanidins, PAs; CCl_4_, carbon tetrachloride

The induction of liver fibrosis by CCl_4_ caused a significant (*P* < 0.05) decrease in anti-apoptotic protein Bcl-2 with a marked increase in pro-apoptotic protein Bax and a significant (*P* < 0.05) increase in the levels of caspase-3 and -9 after 5 and 9 weeks of CCl_4_ intoxication when compared to the healthy rats. The severity of disruption of these apoptotic regulating proteins was higher after 5 weeks than 9 weeks of CCl_4_ treatment. Administration of PAs into CCl_4_-induced rats’ liver fibrosis led to a significant (*P* < 0.05) amelioration at the levels of these apoptotic regulating proteins compared with CCL_4_-treated rats. Treatment with PAs or olive oil alone did not influence the hepatic levels in control rats until treatment (Fig. 7A–D).

### PAs improved DNA destruction in the liver of the fibrosis rat model

**Fig. 8:**
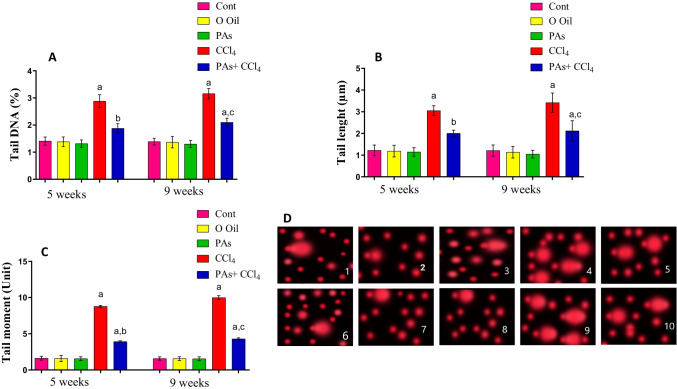
Effect of PAs and CCl_4_ on DNA damage in the liver of control and experimental groups measured by comet assay. (A) tail DNA (%), (B) DNA tail length, (C) tail moment, and (D) representative photomicrographs of comets stained with ethidium bromide at × 200 magnification showing the DNA migration pattern in hepatocytes. (1–5) 5 weeks, (6–10) 9 weeks. (1) Control, (2) olive oil, (3) PAs, (4) CCl_4_, (5) PAS + CCl_4_, (6) Control, (7) olive oil, (8) PAs, (9) CCl_4_, (10) PAS + CCl_4_. CCl_4_-treated rats show extensive DNA migration while concurrent treatment with PAs and CCl_4_-treated rats show minimal DNA migration. Data are expressed as mean ± SEM (*n* = 6 for each group). a, b, c Significant change at *P* < 0.05. a: Significance as compared with control, b: significance as compared with CCl_4_ group in 5 weeks, c: significance as compared with CCl_4_ group in 9 weeks. Cont, control; O oil, olive oil; proanthocyanidins, Pas; CCl_4_, carbon tetrachloride

The DNA damage in the liver was investigated using the comet assay technique. The parameters of comet assay measured, including tail moments (unit), tail length (mm), and percentage of DNA in the tail were significantly (*P* < 0.05) elevated in the liver of CCl_4_-intoxicated rats after 5 and nine weeks, as compared with the normal control rats. In contrast, administration of PAs into CCl_4_-induced rats’ hepatic fibrosis produced a significant reduction in DNA damage, as evidenced by a significant (*P* < 0.05) decrease in the values of these parameters, compared with the CCL_4_-treated rats. Treatment with PAs or olive oil alone showed insignificant (*P* < 0.05) changes in these parameters compared with control rats (Fig. 8A–D).

## Discussion

Liver fibrosis is a major health problem linked with several liver diseases and contributes to a high percentage of global mortality (Roehlen et al. 2020). If it is not prevented, it can develop into cirrhosis, hepatocellular carcinoma, and death (Aydın & Akcali 2018). Oxidative stress and inflammation are the major interdependent mechanisms of liver damage and fibrosis progression. PAs are polyphenolic compounds characterized by several biological effects, including antioxidant and anti-inflammatory effects (Rauf et al. 2019). The interfering with the etiology underlying chronic liver injury-induced fibrosis is the primary goal of anti-fibrotic therapy (Kisseleva and Brenner 2021). Thus, we hypothesized PAs might protect against oxidative damage and improve liver fibrosis through interfering with fibrotic pathways. The present study showed that PAs mitigated CCl4-induced liver damage and suppressed liver fibrosis by inhibiting oxidative stress, ameliorating inflammation, protecting DNA damage, and modulation of apoptotic regulating proteins. This led to the prevention of liver pathology and normalized liver function parameters. The remarkable amelioration of fibro-marker alpha-2-macroglobulin that inhibits proteases and binds several cytokines and growth factors confirmed the fibrosis regression, including transforming growth factor-beta (TGFβ). These findings support a report that proposed a potential therapeutic approaches to hepatic fibrosis by targeting to TGF-β/SMAD signaling (Xu et al. 2016). TGFβ stimulates collagen formation by the human liver and lung myofibroblasts (Tiggelman et al. 1997; Kendall et al. 2021). In addition, PAs prevented fiber formation, as shown by Masson trichrome stain. Thus, it is suggested that PAs are likely to have an anti-fibrotic effect.

The current study showed that CCl_4_ caused liver damage by the elevation of AST, ALT activities, and bilirubin level, and decreased protein and apolipoprotein A1 contents in the serum of CCl_4_-treated rats. This effect is coupled with increased oxidative stress evidenced by increased MDA because of the remarkable decrease in antioxidants in the liver. Lipid peroxidation-induced damage of hepatic cells caused cellular permeability changes and leakage of proteins, including aminotransferases into serum, signifying hepatic dysfunction and necrosis (Yachi et al. 2010). Administration of PAs suppressed hepatic damage and restored liver function evidenced by a significant normalization of hepatic function parameters including AST, ALT activities, bilirubin, apolipoprotein A1, and alpha-2-macroglobulin levels in serum. These findings support a report that proposed a potential therapeutic approaches to hepatic fibrosis by targeting to TGF-β/SMAD signaling (Xu et al. 2016). These findings suggest the ability of PAs to protect hepatocyte membranes against lipid peroxidation-induced membrane injury and leakage of intracellular proteins. This effect might be attributed to the antioxidant properties of PAs and the ability to suppress oxidative stress. The findings that PAs can suppress oxidative stress are confirmed in the present study, as shown by restoring GSH content and GPx and GR activities with a significant decrease in MDA formation in the liver compared with intoxicated rats. The current observation of the antioxidant effect of PAs is compatible with an earlier study that PAs inhibit oxidative stress and liver injury in several situations (Dai et al. 2014; Sun et al. 2018).

Inflammation played an important key role in hepatocyte injury and progression of liver fibrosis (Kisseleva and Brenner 2021). During liver damage, reactive oxygen species (ROS) stimulate the pro-inflammatory genes and overproduction of inflammatory cytokines. The interdependence of oxidative stress and inflammation plays a crucial role in promoting liver fibrosis (Li et al. 2016). The current study showed a significant increase in pro-inflammatory cytokines, including IL-6, IL-1β, and TNF-α, which enhance the attraction and activation of immune cells in the liver of CCl_4_-treated animals. This effect is intensified by lapse of time. PAs prevented the elevation in these cytokine levels, showing an anti-inflammatory effect. The decrease in inflammatory cell infiltration and expression of the pro-inflammatory cytokines might modulate intracellular signaling mechanisms that reduce ROS generation and development of oxidative stress in the liver of CCl_4_-treated rats resulting in suppression of liver injury and fibrosis. Comparable anti-inflammatory effects of PAs are reported earlier (Wang et al. 2015; El-Shitany and Eid 2017). The decrease in pro-inflammatory cytokines (IL-6, IL-1β, and TNF-α) is accompanied by regression of liver fibrosis in CCl_4_-intoxicated rats. The anti-inflammatory effect of PAs was recorded after 5 and 9 weeks of intoxication, showing its anti-inflammatory efficacy.

Apoptosis of injured liver cells induces pathological cascade events, such as inflammation, activation of myofibroblasts, and liver fibrosis (Kisseleva and Brenner 2021). Thus, suppression of hepatocyte apoptosis is a plausible strategy for protecting the liver. Mitochondria are the main organelle that regulates apoptosis because they are the major site for energy production and ROS generation (Abate et al. 2020). To assess the role of PAs in protecting hepatocytes against CCl4-induced apoptosis, we analyzed apoptotic regulating proteins, including Bax, Bcl-2, caspase 3, and caspase 9. It is shown that CCl_4_ caused upregulation of Bax and downregulation of the anti-apoptotic protein Bcl-2 with increased effector caspase-3 and initiator caspase-9 in the liver. This indicates that CCl4 triggered apoptosis via the mitochondrial and death receptor pathways. Administration of PAs effectively prevented CCl_4_-induced activation of apoptotic pathways through modulation of the apoptotic regulating proteins leading to protection of hepatocytes, showing anti-apoptotic effect of PAs. These results are consistent with a previous report that attributed the rescue action on mitochondrial oxidative damage in the liver to the free radical scavenging, metal chelating, and antioxidant potentials of PAs (Miltonprabu et al. 2016). The antioxidant properties of PAs took part in restoring redox balance and anti-apoptotic effect through amelioration of the ROS generation.

The quantification of DNA damage using comet assay revealed that CCl_4_ significantly increased the levels of % DNA in the tail, tail length, and tail movement, showing a significant increase in DNA destruction and hepatocyte apoptosis. Furthermore, treatment of rats with CCl_4_-induced liver fibrosis with PAs caused significant protection against DNA strand breaks, most likely because of radical scavenging function. These findings confirm earlier work that, by declining oxidative stress at an upstream step, PAs efficiently protected against DNA damage in the liver of CCl4-intoxicated rats via CYP2E1 regulation (Dai et al. 2014). This effect most likely represses the induction of apoptosis and carcinogenesis that follow DNA damage. This would further prevent the possibility of incurring irreparable damage and injury to the liver and liver dysfunction (Dai et al. 2014).

To confirm the biochemical observation of the anti-fibrotic effect of PAs, we found that supplementation with PAs interfered with fibrosis formation and exhibited low fibrotic area compared with CCl4-treated rats. Furthermore, the histological features and quantification of fibrosis regression are remarkable after 5 and 9 weeks of intoxication, showing hepatic repair and blocking of cirrhosis induction.

Although Pas’ interfering effect with fibrosis is promising, some issues remain to be explained. The main issues that deserve comprehensive studies are regarding the immunomodulatory effect of PAs and the production of fibrosis-linked growth factors, activation of hepatic myofibroblasts, and excessive production of extracellular molecules. However, after controlled clinical trials, PAs can be used as an adjuvant with conventional chemotherapy.

## Conclusion

In summary, PAs suppressed fibrosis in the liver of CCl_4_-treated rats. Normalized liver function parameters accompany the improvement of liver histological structure. Furthermore, PAs exerted the anti-fibrotic effect by restoring redox balance, suppressing inflammatory cytokines, normalizing apoptotic regulating proteins, and protecting DNA structure. These findings support the medicinal value of PAs for fibrosis-induced diseases.

## Data Availability

All the data generated or analyzed during this study are included in this published article.
